# Transdiagnostic Compulsivity Traits in Problematic Use of the Internet Among UK Residents: Cross-Sectional Network Analysis Study

**DOI:** 10.2196/66191

**Published:** 2025-03-26

**Authors:** Chang Liu, Samuel Chamberlain, Konstantinos Ioannidis, Jeggan Tiego, Jon Grant, Murat Yücel, Peter Hellyer, Christine Lochner, Adam Hampshire, Lucy Albertella

**Affiliations:** 1 School of Psychological Sciences Monash University Clayton Australia; 2 Department of Psychiatry University of Southampton Southampton United Kingdom; 3 Hampshire and Isle of Wight Healthcare NHS Foundation Trust Southampton United Kingdom; 4 Department of Psychiatry and Behavioral Neuroscience University of Chicago Chicago, IL United States; 5 Institute of Psychiatry, Psychology and Neuroscience King's College London London United Kingdom; 6 Department of Psychiatry Stellenbosch University Stellenbosch South Africa; 7 Department of Brain Sciences Imperial College London London United Kingdom

**Keywords:** compulsivity, problematic use of the internet, network analysis, perfectionism, reward drive, cognitive rigidity, transdiagnostic, PUI, mental health, intrapersonal factor, cognitive, internet use, network

## Abstract

**Background:**

The societal and public health costs of problematic use of the internet (PUI) are increasingly recognized as a concern across all age groups, presenting a growing challenge for mental health research. International scientific initiatives have emphasized the need to explore the potential roles of personality features in PUI. Compulsivity is a key personality trait associated with PUI and has been recognized by experts as a critical factor that should be prioritized in PUI research. Given that compulsivity is a multidimensional construct and PUI encompasses diverse symptoms, different underlying mechanisms are likely involved. However, the specific relationships between compulsivity dimensions and PUI symptoms remain unclear, limiting our understanding of compulsivity’s role in PUI.

**Objective:**

This study aimed to clarify the unique relationships among different dimensions of compulsivity, namely, perfectionism, reward drive, cognitive rigidity, and symptoms of PUI using a symptom-based network approach.

**Methods:**

A regularized partial-correlation network was fitted using a large-scale sample from the United Kingdom. Bridge centrality analysis was conducted to identify bridge nodes within the network. Node predictability analysis was performed to assess the self-determination and controllability of the nodes within the network.

**Results:**

The sample comprised 122,345 individuals from the United Kingdom (51.4% female, age: mean 43.7, SD 16.5, range 9-86 years). The analysis identified several strong mechanistic relationships. The strongest positive intracluster edge was between reward drive and PUI4 (financial consequences due to internet use; weight=0.11). Meanwhile, the strongest negative intracluster edge was between perfectionism and PUI4 (financial consequences due to internet use; weight=0.04). Cognitive rigidity showed strong relationships with PUI2 (internet use for distress relief; weight=0.06) and PUI3 (internet use for loneliness or boredom; weight=0.07). Notably, reward drive (bridge expected influence=0.32) and cognitive rigidity (bridge expected influence=0.16) were identified as key bridge nodes, positively associated with PUI symptoms. Meanwhile, perfectionism exhibited a negative association with PUI symptoms (bridge expected influence=–0.05). The network’s overall mean predictability was 0.37, with PUI6 (compulsion, predictability=0.55) showing the highest predictability.

**Conclusions:**

The findings reveal distinct relationships between different dimensions of compulsivity and individual PUI symptoms, supporting the importance of choosing targeted interventions based on individual symptom profiles. In addition, the identified bridge nodes, reward drive, and cognitive rigidity may represent promising targets for PUI prevention and intervention and warrant further investigation.

## Introduction

Problematic use of the internet (PUI) is characterized by excessive and maladaptive internet use that leads to significant functional impairment and distress [1]. PUI encompasses a variety of potentially harmful online behaviors such (but not limited to) as excessive gaming, viewing pornography, social networking, and streaming content consumption [2]. It is recognized as a global public health concern, receiving increasing attention from mental health researchers, clinicians and policy makers [1,2]. A recent meta-analysis showed that the rate of PUI is estimated to be 14.2% globally [3], though estimates vary depending on the operational definition of PUI. Mental and physical impairments associated with PUI are well-documented, including depression, anxiety, musculoskeletal problems, and digital eye strain [4-7]. Given the high rates and associated impairments of PUI, it is important to understand the underpinning mechanisms.

Clarifying the role of intrapersonal risk factors (eg, personality traits) in PUI has been identified as a research priority for understanding PUI [1,2]. Compulsivity is defined as the tendency toward repetitive actions that persist despite such actions being inconsistent with one’s overall goal and associated negative consequences [8,9]. It is considered a hallmark feature of addictions and obsessive-compulsive disorder [10] and has been consistently linked to PUI [11-14]. For instance, recent evidence suggests that compulsivity may be more closely related to PUI than other well-established risk factors (ie, impulsivity traits [15]).

Compulsivity is oftentimes treated as a unitary construct when estimating its relationships with PUI. However, recent evidence suggests that compulsivity can be decomposed into various distinct but interrelated dimensions. Specifically, a large-scale network-based study [16] has shown that compulsivity may be composed of 3 dimensions: perfectionism (having high personal standards and striving to reach those standards), reward drive (approach tendencies toward immediate gratification and acting on urges) and cognitive rigidity (rigid and repetitive thinking patterns and behaviors). These distinct dimensions of compulsivity likely have different relationships with PUI. For instance, individuals high on reward drive may be more driven to use the internet for its rewarding features (eg, obtain social rewards, online gambling, and cybersex [17-19]). Alternatively, individuals high in cognitive rigidity may find it harder to redirect their attention away from internet-related cues and thoughts and may be prone to activities such as checking the internet due to health-related anxieties (ie, cyberchondria [20]). Finally, individuals high in perfectionism may be inclined to use the internet as a way to cope with the discrepancy between their ideal and actual life achievements [21]. Identifying how different compulsivity dimensions are differently linked with PUI may offer important insights into the mechanistic relationships between compulsivity and PUI.

Further, there is a lack of understanding of the specific symptom pathways between compulsivity dimensions and PUI. Using symptom sum scores as an index for PUI is a common practice when estimating the relationship between compulsivity and PUI. However, recent studies have demonstrated that PUI symptoms differ from each other in their relation to risk factors [15,22,23]. For instance, it has been found that compulsivity is more closely related to PUI symptoms characterized by negative consequences (eg, failure to fulfil role obligations [15]) than other PUI symptoms, such as staying online longer than intended, hiding online time from others or acting aggressively when being disrupted during online time. In addition, it has been observed that individuals with PUI symptoms characterized by continued or escalated usage report experiencing less fear of missing out [24]. Understanding these more specific relations may facilitate a more precise understanding of specific mechanisms underlying different PUI symptom profiles.

A network approach may be particularly useful to address the aforementioned issues and provide a more precise understanding of the relations between different dimensions of compulsivity and symptoms of PUI. The network approach to psychopathology posits that mental conditions can be conceptualized as a complex network, constituted by a set of nodes (eg, item-level characteristics and symptoms) and edges that connect them [25,26]. Accordingly, the associated analytic techniques focus on pinpointing specific and distinct relationships between nodes (eg, dimensions of compulsivity and symptoms of PUI) and visualizing these relationships in insightful ways [27]. Hence, the utilization of the network approach moves beyond examining how compulsivity traits may be associated with the PUI construct (indexed by the symptom sum score) to elucidate differential relationships between compulsivity dimensions and specific symptoms of PUI.

It is worth noting that the network approach also provides novel indices to identify influential nodes that may serve as targets of therapeutic intervention. The bridge expected influence calculates the sum of connectivity of a given node to all nodes of the other cluster (a subset of nodes in the network with denser connections among themselves, forming a subnetwork [28]). Nodes that exhibit high bridge centrality (bridge nodes) are deemed to be integral to the co-occurrence of constructs, given their extensive interconnectivity with all components of the other constructs [25]. Theoretically, targeting such nodes has the potential to impede the transmission of activation from one construct to another [25,29]. Accordingly, it has been proposed that nodes with high bridge centrality may be ideal candidates for intervention. Given that PUI treatments (both psychological and pharmacological) are still in their infancy, such understanding is both timely and critical to push this field forward.

There is a paucity of research on the relationship between dimensions of compulsivity and PUI symptoms. The current study is the first attempt to fill the knowledge gap using a symptom-based network approach. By investigating the network structure and bridge centrality index, the current study aimed to (1) elucidate the distinct relationships between dimensions of compulsivity (ie, perfectionism, reward drive, and cognitive rigidity) and PUI symptoms, (2) quantify the extent to which each dimension of compulsivity is related to the PUI symptom cluster and identify bridge nodes, and (3) examine the controllability of each node within the network.

## Methods

### Participants and Procedure

The sample included 122,345 individuals (51.4% female) from the United Kingdom, with a mean age of 43.7 (SD 16.5, range 9-86) years, who participated in the online Great British Intelligence Test (GBIT). The GBIT is a collaborative citizen science project with BBC two Horizon Program, designed to investigate cognition and well-being in the general population. The study was an open, web-based survey using convenience sampling, accessible to all UK residents. It was promoted across BBC media channels, both online and offline, to recruit participants. Interested individuals could access the study through links provided in mainstream media and news articles, which directed them to the study’s landing page on the Cognition website. The recruitment for the GBIT study commenced in December 2019, with the specific sample used in this paper recruited between May and June 2020. For the current study, we focused solely on individuals who provided complete responses for both measures. The compulsivity measure was introduced during a later phase of the GBIT. Among the 122,680 participants who took part after the compulsivity measure was included, only a small proportion of responses were missing, specifically, 335 for the compulsivity measure and 45 for PUI items. These incomplete responses were excluded from the data analysis.

### Measures

#### Trait Compulsivity

The Cambridge-Chicago Compulsivity Trait Scale (CHI-T; [30]) was used to measure trait compulsivity. The CHI-T contains 15 items, which cover 3 dimensions of compulsivity (perfectionism, cognitive rigidity and reward-drive; [16]). The perfectionism subscale covers self-oriented perfectionism, which features having high personal standards and striving for perfection and “just right” (eg, “completion to a high standard” and “doing things just right”). The cognitive rigidity subscale covers the inability to change behaviors or mindset (eg, “repetitive thoughts” and “being stubborn or rigid”). The reward drive subscale covers approach tendencies toward immediate gratification and acting on urges (eg, “acting on urges” and “seeking immediate reward”). We have opted for the subscale score over the item score due to practical considerations. Currently, interventions for individual items of the CHI-T scale are undeveloped. In contrast, targeted therapies for specific subscales, like attentional bias modification therapy, already exist and may effectively reduce reward-drive tendencies. Therefore, using the subscale score may enhance the applicability of our findings in future intervention development.

#### PUI Symptoms

PUI symptoms were measured by 7 items based on expert consensus (Hampshire et al [31]). The items measure internet checking, internet use for distress relief, internet use for loneliness or boredom, financial consequences due to internet use, prioritizing internet use over role obligations, compulsion and failed attempts to cut down, in the past 4 weeks. Response options range from 0 (never) to 6 (more than hourly every day).

### Data Analysis

Descriptive statistics were calculated for demographic variables, CHI-T subscales, and PUI items. Before conducting our formal analysis, we used the goldbricker function to check for significant topological overlap among nodes, which identifies pairs with highly similar connectivity patterns, indicative of duplicates [32]. Specifically, we looked for node pairs with a topological overlap exceeding 80%, which corresponds to less than 20% difference in their connectivity patterns (significant at *P*<.01). No topological overlap was identified (nor was it for the detailed item-level analysis in the supplement). We constructed a regularized partial correlation network, where the variables (ie, the 3 dimensions of compulsivity and individual PUI symptoms) are represented as nodes. The edges between nodes represent regularized partial correlations, controlling for all other variables within the network. The Graphical Least Absolute Shrinkage and Selection Operator algorithm was applied for regularization [33]. The regularization procedure forces weak or spurious partial correlations to become exactly zero, leading to a sparse network where only the most robust edges are retained [33]. Following recommendations from [33], we set the tuning parameter (γ) for regularization to 0.5, which balances the trade-off between model fit and sparsity of the network.

The Fruchterman-Reingold layout algorithm was used for network visualization [34]. Within each network, strongly related nodes were placed closer together, while weakly related nodes were placed further apart. The magnitudes of partial correlations (edge weights) are reflected as edge thickness, with thicker edges indicating stronger partial correlations between nodes. Blue edges represent positive partial correlations, and red edges represent negative partial correlations between nodes. The visualization procedures were conducted via the “qgraph” package in R (R Core Team) [34]. To enhance visual clarity, we adjusted the scaling of edge widths and color saturation using the cut argument. The improved visual representation of the network is available (Figure S5 in Multimedia Appendix 1).

We used the “networktools” package [32] to quantify to what extent each compulsivity dimension was related to the PUI symptom cluster and identify influential bridge nodes. Number of 2 clusters (subnetworks) were predefined for the network, namely, the compulsivity cluster (consisting of 3 compulsivity dimensions) and the PUI community (consisting of PUI items). Bridge expected influence (ie, the sum of the edge weights connecting a given node to all nodes in the other cluster; [25] was computed for each node. The compulsivity dimension with a high positive bridge expected influence is positively related to the PUI symptom cluster. Conversely, the compulsivity dimension with a negative bridge expected influence is negatively related to the PUI symptom cluster.

We also examined the predictability of nodes, assessing how well a specific node can be predicted by all other nodes within the network [35,36]. Since all the variables included are continuous, we selected *R*² as the measure of predictability for the estimated network. A node with higher predictability suggests that a larger proportion of its variance can be explained by other nodes in the network [35]. Further, we also computed the mean predictability across nodes within the network. This allows us to determine whether, on average, the variance observed in the nodes of the network can be explained by other nodes within the network [35,36]. A high mean predictability indicates that a network is largely determined by internal nodes within itself [35,36].

To estimate the accuracy and stability of the network model, we followed the steps described in [37]. First, we bootstrapped 95% CIs (with 10,000 bootstrap samples) of edge weights to determine the accuracy of the estimated edges within each network. Second, we estimated the stability of edges and bridge centrality using the case-dropping bootstrap procedure (with 10,000 bootstrap samples) and calculated the correlation stability coefficient (CS-coefficient) for edge weight and bridge expected influence. An acceptable CS-coefficient should be above 0.25, and a value of 0.5 or higher indicates optimal stability. Finally, in alignment with the tutorial paper [37], we examined whether the weights for each edge and the bridge centrality estimates for each node differ significantly (α=.05) from one another. This was achieved using bootstrapped difference tests for edge weights and bridge centrality indices. The “bootnet” package was used to perform the accuracy and stability tests [38].

We acknowledge that the item-level findings may add further nuance to the current results. To offer a comprehensive overview, we have included the item-level network analysis in the for readers interested in detailed item-level analysis (Figure S6 in Multimedia Appendix 1).

### Ethical Considerations

The study adhered to the ethical principles for medical research involving human subjects outlined in the Helsinki Declaration of 1975, as revised in 2008, and received approval from the institutional review board at Imperial College London (approval number 17IC4009). All participants were fully informed about the study details and provided their informed consent before participation. All data collected were anonymous. Participation was voluntary, and no financial incentives were offered.

## Results

Table 1 presents demographic characteristics of the study sample. Descriptive statistics for CHI-T subscale scores and PUI symptom scores are provided (Table S1 in Multimedia Appendix 1). The clinical characteristics of the sample are presented (Table S2 in Multimedia Appendix 1).

**Figure 1:**
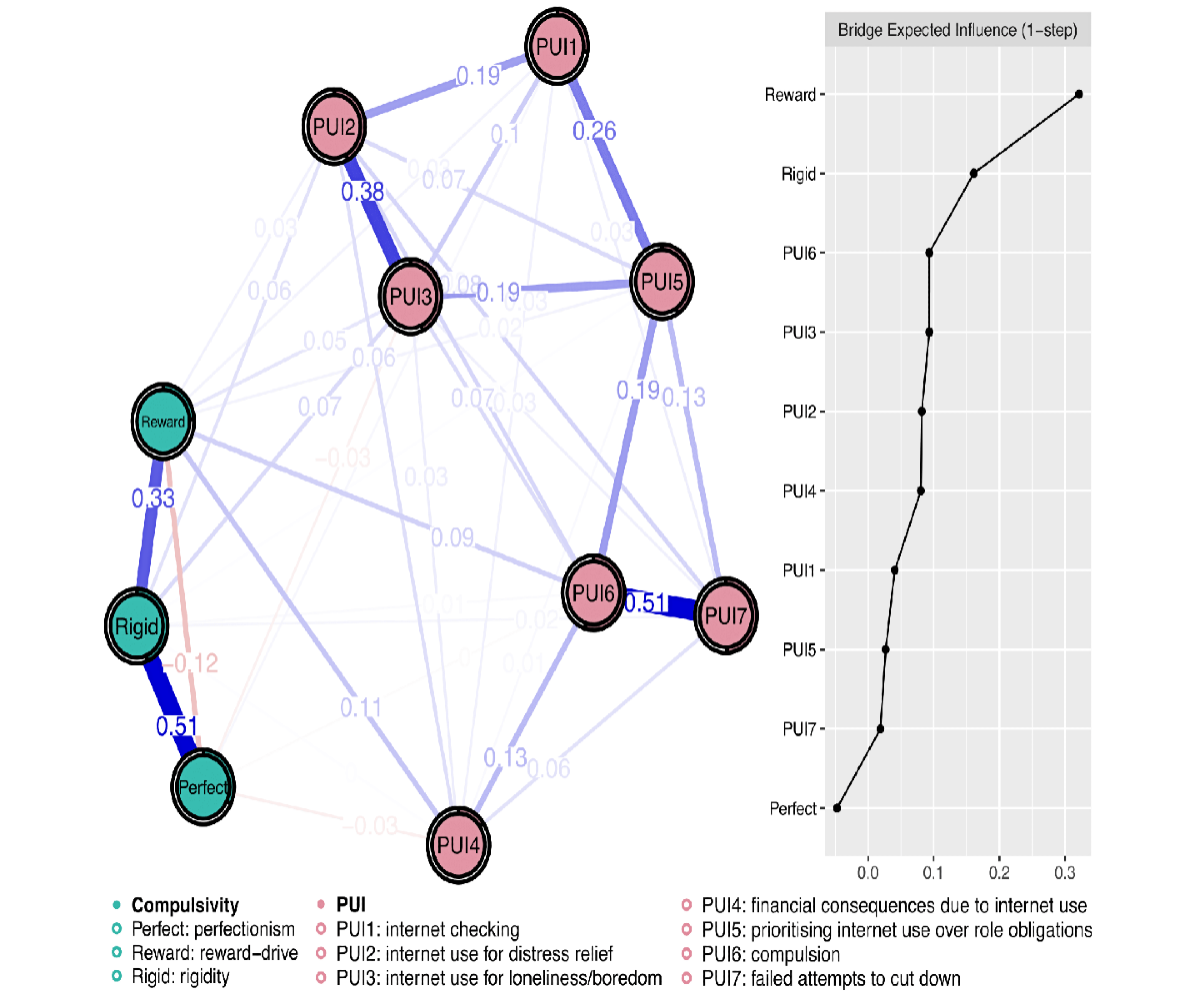
Compulsivity-PUI network and bridge centrality plot of UK residents who participated in the online GBIT and completed compulsivity and PUI measures between May and June 2020.

Figure 1 presents the estimated compulsivity-PUI network and the bridge centrality plot (ie, raw bridge expected values of each node within the network). The network was densely connected, with 39 remaining edges (out of 45 possible edges). The strongest positive intracluster edge emerged between reward-drive and PUI4 (financial consequences due to internet use; edge weight=0.11). The strongest negative intracluster edge emerged between perfectionism and PUI4 (financial consequences due to internet use; edge weight=–0.04). Cognitive rigidity was strongly associated with PUI2 (internet use for distress relief; edge weight=0.06) and PUI3 (internet use for loneliness or boredom; edge weight=0.07). We found that reward-drive had the highest positive bridge expected influence, while perfectionism had the highest negative bridge expected influence.

The predictability of nodes was plotted as the colored area in the ring around each node. The nodes exhibiting the highest predictability were PUI6 (compulsion, predictability=0.55); PUI7 (failed attempts to cut down, predictability=0.49), and PUI3 (internet use for loneliness or boredom, predictability=0.44). The network’s overall mean predictability is 0.37, which is comparable to the previously reported average level of predictability (mean predictability=0.32) in the literature [35]. The raw predictability scores can be found (Table S3 in Multimedia Appendix 1).

The narrow confidence intervals for edge weights (Figure S1) indicate that estimated edges within networks were accurate. The CS-coefficients for bridge expected influence were excellent in the estimated network (value=0.75), indicating optimal stability for the bridge centrality index. Results from the bootstrapped stability and accuracy tests are included (Figure S1 in Multimedia Appendix 1 and Figure S3 in Multimedia Appendix 1). The results of bootstrapped difference tests are included (Figure S2 in Multimedia Appendix 1 and Figure S4 in Multimedia Appendix 1).

**Table 1:** Descriptive statistics of demographic variables for UK residents who participated in the online Great British Intelligence Test and completed the problematic use of the internet and compulsivity measures between May and June 2020.

Variable				Values			
Age (years), mean (SD)				43.7 (16.5)			
**Sex, n (%)**							
			Female			62286 (51.4)	
			Male			58728 (48)	
			Nonbinary/other			710 (0.6)	
**Education level, n (%)**							
		No schooling			290 (0.2)		
		Primary school			2592 (2.1)		
		High school or diploma			41469 (33.9)		
		Bachelor’s or master’s degree			68644 (54.1)		
		Doctoral degree			4621 (3.8)		
		Other			4729 (3.9)		
**Ethnicity, n (%)**							
	Mixed ethnicity						3367 (2.8)
	North African						260 (0.2)
	Rom, Sinti or Bedouin						100 (0.1)
	Sub-Saharan African or Afro-American						551 (0.5)
	West-central Asian						499 (0.4)
	White						108497 (88.7)
	Nondisclosure						9071 (7.4)

## Discussion

### Principal Findings

This study is the first and largest to date to explore the relationships between different dimensions of compulsivity and individual PUI symptoms through network analysis. Extensive intercluster connections were found, indicating that compulsivity and PUI were strongly interrelated. We identified several strong mechanistic pathways, notably the edge between cognitive rigidity and coping and the edge between reward drive and functional impairment (ie, negative financial consequences). Finally, reward drive and cognitive rigidity were identified as the bridge nodes.

When elucidating interrelationships between compulsivity dimensions and PUI symptoms, we found that cognitive rigidity was associated with coping-motivated internet use. The results are in line with previous research [39-41], which highlights the role of rigid coping tendencies as a core mechanism underlying the relationship between compulsivity and addictive behaviors. Specifically, individuals high on cognitive rigidity may have a narrowed coping repertoire when experiencing negative emotions that, in turn, limits engagement in alternative coping strategies [42]. Several features of internet use (eg, easy accessibility and providing immediate stress relief) make it easy for individuals to use it as a coping strategy. However, such a coping strategy may be counterproductive in the long run (eg, due to excessive time spent online and avoiding dealing with daily challenges). For individuals with high cognitive rigidity, the inability to generate and adopt alternative coping responses when they need these most (ie, under stress) may result in their persistent engagement in coping-motivated internet use, even if this coping strategy proves to be ineffective and impairing.

Another strong pathway emerged between reward drive and functional impairment (ie, negative financial consequences and sleep loss), which may be explained by the counterproductive attentional capture mechanism characteristic of compulsivity [11,42]. Specifically, individuals with high reward drive may exhibit stronger attraction toward reward-related cues, finding it difficult to resist approaching these cues even when doing so may be counterproductive. In these individuals, such approach tendencies could be triggered by reward-related stimuli presented on the internet (eg, applications and websites for recreational online gaming, gambling, and cybersex), leading to internet use despite such use not being in line with an individual’s goals and resulting in negative consequences.

Interestingly, negative relationships were observed between perfectionism and PUI symptoms. The relationship between perfectionism and addictive behaviors tends to be inconsistent across studies, with some studies finding that perfectionism may actually protect individuals from addiction-related impairments [43] and problematic drinking [44]. This inconsistency may stem from variations in how perfectionism is measured. Perfectionism is considered as a multidimensional construct, encompassing types like self-oriented, other-oriented, and socially prescribed perfectionism [45]. In this study, the perfectionism dimension captures self-oriented perfectionism (ie, holding high standards and striving for perfection or “just right”). It has been posited that specific aspects of perfectionism (ie, self-oriented perfectionism) may be considered a form of hyperconscientiousness [44]. Such hyper-conscientiousness may protect individuals from particular addictive behaviors, which are considered impairing and not aligned with their goals (eg, being perfect in everything; [44]). Our results provide support for this hypothesis by showing that perfectionism was negatively associated with self-report items measuring functional impairment.

The bridge centrality analysis identified reward drive and cognitive rigidity as bridge nodes that positively relate to the PUI symptom cluster, whereas perfectionism negatively relates to it. The roles of reward drive and cognitive rigidity in PUI are in line with previous studies that have demonstrated attentional approach tendencies toward reward cues and rigidity of such attentional responses are linked to obsessive-compulsive and addictive behaviors, including PUI [11,41]. Meanwhile, by showing that perfectionism is negatively related to the PUI symptom cluster, our findings extend previous assumptions on the potential protective role of perfectionism (underpinned by conscientiousness) in problematic alcohol use [44] to suggest that such a protective effect may be transdiagnostic across substance use and behavioral addictions.

Current findings addressed several key research priorities identified by experts [1,2]. First, our findings help elucidate the relations between compulsivity dimensions and PUI symptoms, which addressed the need for clarifying the role of compulsivity in PUI [1,2]. The distinct connectivity between traits and symptoms may suggest that interventions should be personalized to address individual symptom profiles. For example, interventions might focus on reducing reward-driven tendencies rather than targeting perfectionism in individuals whose problematic usage is characterized by financial consequences, to achieve better intervention outcomes. Second, by demonstrating the role of reward drive and cognitive rigidity, our results may inform efforts to identify at-risk individuals at the earliest stages of PUI and provide early intervention [2]. Specifically, given that compulsivity traits can exist before the emergence of PUI symptoms, reward drive and cognitive rigidity traits should, in future work, be evaluated in terms of their potential value in identifying individuals who are most at risk of developing PUI. In turn, this may inform our screening processes.

Previous simulation research on the role of bridge nodes has demonstrated that “focusing treatment on the detected nodes is effective in preventing the contagion of comorbid disorders” ([25], P. 365). Empirically, if we can establish that the identified bridge nodes, namely reward drive and cognitive rigidity, exhibit temporal precedence, these findings could mark a significant step toward intervention target selection.

Finally, node predictability may shed several practical insights. For instance, using the internet to cope with loneliness or boredom was identified as one of the nodes with the highest predictability. The predictability allows an understanding of the clinical relevance of the identified mechanism. For instance, we found that a significant proportion of variance in using the internet to cope with loneliness or boredom may be explained by its neighboring nodes (reward-drive and cognitive rigidity). This may indicate that intervention on reward drive and cognitive rigidity may have a higher success potential in reducing coping-motivated internet use. Further, the mean predictability of the network suggests that although compulsivity dimensions may play significant roles in PUI, other factors may also be involved in driving PUI and should be considered when planning interventions.

Based on our findings, targeting reward drive and cognitive rigidity (the bridge nodes) via digital personality change interventions [46] may be expected to improve treatment outcomes. Specifically, digital personality change interventions incorporate evidence-based techniques that foster and sustain personality change (eg, tailored implementation intentions (if-then plans), psychoeducation, behavioral activation, self-reflection, resource activation, and individual progress feedback), alongside a chatbot that facilitates coaching dialogues to simulate a personal coaching process [47]. A large-scale randomized controlled trial (n=1523) has shown that a digital personality change intervention was associated with significant changes in big 5 personality traits, versus waiting list control, with effects persisting beyond the intervention [47]. The promising findings suggest that such interventions have promise in potentially improving compulsivity traits such as reward drive and cognitive rigidity. It should be noted that the aforementioned study did not directly measure compulsivity, but rather traits associated with it (eg, conscientiousness), as classic personality inventories typically do not assess compulsivity. Future studies may consider directly examining the extent to which the intervention can effectively reduce dimensions of compulsivity.

Considering the transdiagnostic characteristics of mechanisms discussed above (ie, counterproductive attentional capture, rigid coping styles, and conscientiousness), such interventions may potentially help reduce other compulsive behaviors (eg, problematic drinking, binge-eating, and obsessive-compulsive behaviors) that frequently co-occur with PUI [48]. These suggestions should be tested in future research studies.

### Limitations

Several limitations should be noted when interpreting current findings. First, PUI symptoms in the current study were measured using items drawn from expert consensus, which may raise concerns regarding the psychometric properties of these items. This was the case for pragmatic reasons as the data were collected as part of a citizen science study [31] that needed to have relatively brief questions that were also acceptable to other stakeholders. The advantage of this approach is that it enables a very large sample size to be collected. Second, the findings were analyzed and discussed with the theoretical assumption that personality traits act as predisposing factors and predict PUI (instead of the other way around; [49-52]. However, due to the cross-sectional nature of the study, we cannot disregard the possibility that PUI may contribute to compulsive traits. It will be important for future research to test the temporal precedence of compulsivity traits and PUI symptoms with longitudinal network analysis. Third, the majority of the sample self-identified as White or Caucasian and resided in a Western country (ie, the United Kingdom), potentially limiting the generalizability of these findings to individuals from other ethnic or cultural backgrounds. Fourth, the current findings were based on data from the general population, which may not be generalizable to individuals with severe levels of PUI. It is important for future research to replicate current findings among individuals with severe levels of PUI.

### Future Directions

In theory, targeting bridge nodes could limit the transmission of activation between different psychological symptoms (or conditions), potentially preventing the onset of comorbid psychopathologies and thus making them valuable targets for intervention [25]. However, empirical evidence on the predictive validity of bridge nodes is inconsistent. For instance, findings from 6 randomized control trials suggest that bridge nodes may predict treatment outcomes in patients with depressive disorders [53]. In addition, studies indicate that bridge nodes can statistically predict the persistence of psychopathology, such as poorer psychosocial functioning and lower BMI in adolescents with anorexia nervosa [54], as well as the future onset of anxiety disorders [55]. Conversely, some evidence suggests that bridge nodes might only partially predict treatment outcomes (ie, predict weight restoration, but not full remission, in patients with anorexia nervosa [56]) or yield nonsignificant results in terms of relationships to outcomes [57]. Given these mixed results, future longitudinal and intervention research is essential to ascertain the predictive validity of bridge nodes before they can be confidently used as treatment targets.

Most research in the fields of psychology and psychiatry that uses network-based approaches has relied on cross-sectional, between-subject designs. However, there is an increasing shift toward using longitudinal designs, including panel data and intensive time-series data [58-60]. These methods enable researchers to differentiate within-subject and between-subject effects and to elucidate the temporal dynamics among psychological constructs. A recent study using graphical vector autoregressive network models to analyze panel data revealed that coping-motivated substance use could predict posttraumatic stress disorder symptoms over time [59]. In addition, when combined with intensive time-series data, graphical vector autoregressive network models can elucidate the interrelationships among psychological symptoms and the impact of risk factors on these symptoms at an individual level, offering insights into precision medicine (eg, by enabling personalized diagnostic profiling and treatment planning; [58,61]). To improve the clinical relevance of current findings, future studies should use longitudinal designs to further explore the network structure and bridge nodes identified in this study.

### Conclusions

The current study addresses evidence gaps in the literature by investigating the relations between different dimensions of self-reported compulsivity and PUI symptoms. using network analysis, we identified distinct connections between dimensions of compulsivity and PUI symptoms. Specifically, we found that reward-drive and cognitive rigidity were differently associated with PUI symptom clusters, which may reflect distinct mechanisms (ie, counterproductive attentional capture and rigid coping response). Further, we identified “reward drive” and “cognitive rigidity” as bridge nodes within the network. These findings represent an initial exploration into the role of transdiagnostic compulsivity in developing future interventions for PUI. Examining the direction of the mechanistic pathways identified, whether compulsivity drives PUI or vice versa, through longitudinal studies represents a promising next step for applying network-informed treatment strategies in clinical practice for PUI.

## Appendix

Multimedia Appendix 1 The results of the Cambridge-Chicago Compulsivity Trait Scale–problematic use of the internet network.

## Abbreviations

CD-coefficient: correlation stability coefficient
CHI-T: Cambridge-Chicago Compulsivity Trait Scale
GBIT: Great British Intelligence Test
PUI: problematic use of the internet

## Data Availability

Data sharing requests from researchers within academic institutions should be directed to Adam Hampshire.
