# Supplementary material for: Transdiagnostic Compulsivity Traits in Problematic Use of the Internet Among UK Residents: Cross-Sectional Network Analysis Study
Source: J Med Internet Res. 2025 Mar 26;27:e66191. doi: 10.2196/66191 (PMC11982749; doi:10.2196/66191)
Supplement: Multimedia Appendix 1 [file jmir_v27i1e66191_app1.docx]

**Supplementary Materials**

**The results of the CHI-T-PUI network**

1. Figure S1. Accuracy of edge weights
2. Figure S2. Bootstrapped difference test for edge weights
3. Figure S3. Stability of bridge expected influences
4. Figure S4. Bootstrapped difference test for bridge expected influences
5. Figure S5. The Compulsivity-PUI network with the cut-argument
6. Figure S6. The item-level Compulsivity-PUI network
7. Table S1. Descriptive statistics of measured variables
8. Table S2. Predictability of variables
9. Table S3. Clinical characteristics of the sample

Figure S1. Accuracy of edge weights. The red line depicts the sample edge weights and the gray bar depicts the bootstrapped confidence interval.

Figure S2. Bootstrapped difference test for edge weights. Gray boxes indicate edge weights that do not differ significantly (*α* = 0.05) from one another, while black boxes indicate edge weights that do differ significantly. Blue and red boxes on the diagonal correspond to edge weights with positive and negative correlations, respectively.

Figure S3. Bootstrapped difference test for bridge expected influences. Gray boxes represent nodes that do not differ significantly from each other (α = 0.05), while black boxes indicate nodes that do differ significantly. White boxes display the bridge expected influences for each node.

Figure S4. Stability of bridge expected influences. The red bar represents the average correlation between bridge expected influences in the full sample and subsample with the red area depicting the 2.5th quantile to the 97.5th quantile.


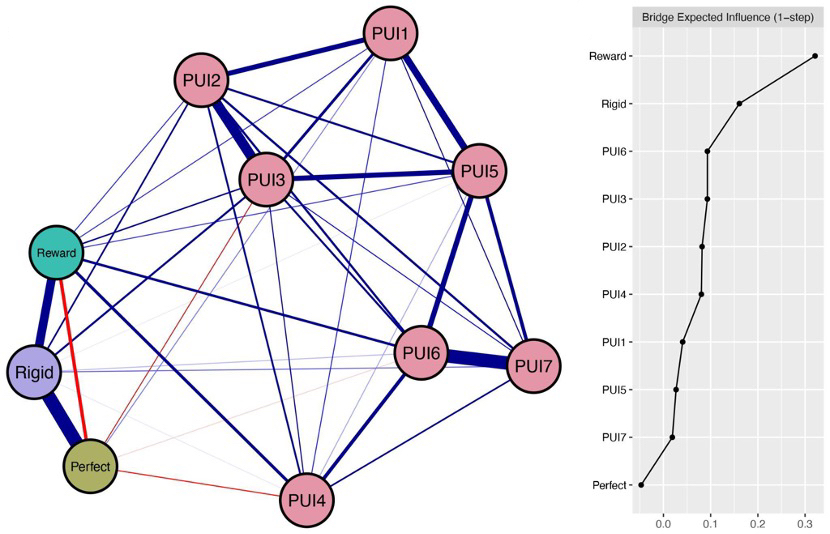


Figure S5. The Compulsivity-PUI network and bridge centrality plot of UK residents who participated in the online GBIT and completed compulsivity and PUI measures between May and June 2020. Cut value = 0.03.


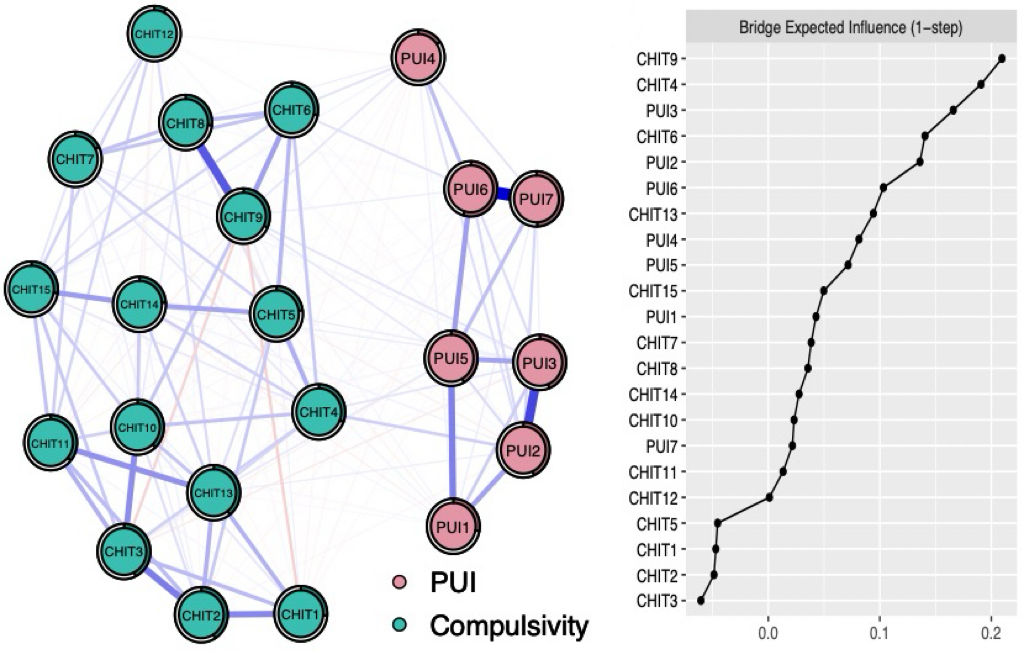


Figure S6. The item-level Compulsivity-PUI network and bridge centrality plot of UK residents who participated in the online GBIT and completed compulsivity and PUI measures between May and June 2020.

Table S1. Descriptive statistics of variables

| Variable | *Mean* | *SD* |
| --- | --- | --- |
|  |  |  |
| 1. PUI1 (How often did you check email or social media accounts after you went to bed?) | 1.47 | 1.54 |
|  |  |  |
| 2. PUI2 (How often did you use internet related activities to block out disturbing thoughts or soothe yourself?) | 1.39 | 1.51 |
|  |  |  |
|  |  |  |
| 3. PUI3 (How often did you choose to spend time on internet related activities to battle loneliness or boredom?) | 2.09 | 1.64 |
|  |  |  |
|  |  |  |
| 4. PUI4 (How often did you suffer from negative financial consequences because of an  online activity?) | 0.24 | 0.56 |
|  |  |  |
|  |  |  |
| 5. PUI5 (How often did you check your email or social media account or equivalent before something else that you needed to do?) | 1.97 | 1.52 |
|  |  |  |
|  |  |  |
| 6. PUI6 (How often did you try to stop an excessive online activity but feel a compulsion to continue?) | 1.06 | 1.29 |
|  |  |  |
|  |  |  |
| 7. PUI7 (How often did you try to cut down the amount of time you spend on-line and fail?) | 0.90 | 1.13 |
|  |  |  |
|  |  |  |
| 9. Reward Drive | 1.88 | 0.95 |
|  |  |  |
|  |  |  |
| 9. Perfectionism | 2.74 | 0.79 |
|  |  |  |
|  |  |  |
| 10. Cognitive Rigidity | 2.26 | 0.69 |
|  |  |  |
|  |  |  |

Table S2. Predictability of variables

| Variable | Predictability |
| --- | --- |
| PUI1 (How often did you check email or social media accounts after you went to bed?) | 0.28 |
| PUI2 (How often did you use internet related activities to block out disturbing thoughts or soothe yourself?) | 0.43 |
| PUI3 (How often did you choose to spend time on internet related activities to battle loneliness or boredom?) | 0.44 |
| PUI4 (How often did you suffer from negative financial consequences because of an online activity?) | 0.15 |
| PUI5 (How often did you check your email or social media account or equivalent before something else that you needed to do?) | 0.41 |
| PUI6 (How often did you try to stop an excessive online activity but feel a compulsion to continue?) | 0.55 |
| PUI7 (How often did you try to cut down the amount of time you spend on-line and fail?) | 0.49 |
| Reward Drive | 0.24 |
| Perfectionism | 0.33 |
| Cognitive Rigidity | 0.44 |

Table S3. Clinical characteristics of the sample

| Variable | *N (percentage)* |
| --- | --- |
| Anxiety | 11,606 (9.5) |
| Attention-deficit/hyperactivity disorder | 540 (.4) |
| Bipolar | 396 (.3) |
| Depression | 11,576 (9.5) |
| Obsessive-compulsive disorder | 947 (.8) |
| Learning disability | 975 (.8) |
| Dementia | 65 (.1) |
| Multiple sclerosis | 290 (.2) |
| Stroke | 499 (.4) |
| Traumatic brain injury | 113 (.1) |
| Parkinson | 164 (.1) |
